# Human parvovirus B19 infection in a pregnant patient resulting in severe hydrops, foetal death and persistent infection

**DOI:** 10.1099/acmi.0.000428

**Published:** 2022-10-03

**Authors:** Ariel Bertoldi, María Belén Colazo Salbetti, Gonzalo Rodríguez, Magdalena Tenaglia, Gabriela Hernández, Jimena Alfaro, María Inés Riberi, Nicolás Lionel Olivera, Mauro Pedranti, María Beatriz Isa, Maria Pilar Adamo

**Affiliations:** ^1^​ Clínica Universitaria Reina Fabiola, Córdoba, Argentina; ^2^​ Instituto de Virología “Dr. J. M. Vanella”, Facultad de Ciencias Médicas, Universidad Nacional de Córdoba, Córdoba, Argentina; ^3^​ Laboratorio de Hemoderivados, Universidad Nacional de Córdoba, Córdoba, Argentina

**Keywords:** congenital diagnosis, congenital hypoplastic anemia, non-immune *hydrops fetalis*, serological tests, Viremia

## Abstract

Human parvovirus B19 (B19V) is the aetiological agent of *erythema infectiosum*. Primary infection during pregnancy can be transmitted to the foetus and cause foetal abnormalities related to depletion of erythrocyte progenitor cells, including congenital anaemia, hydrops, and foetal death. In this paper we report the detection of B19V infection in a pregnant patient, which onset occurred without appreciable signs and symptoms until she developed inappropriate contractions for gestational age and fluid loss. B19V infection resulted in severe *hydrops fetalis* with a fatal course for the foetus, while persisted in the mother at least 12 months after foetal death. The objective of this report is to highlight the importance of optimizing B19V diagnosis through early suspicion and testing during pregnancy. Knowing the mother’s immune status before or at the beginning of gestation can contribute, together with early diagnosis, to improve the management of patients at risk.

## Introduction

Human parvovirus B19 (B19V, officially named *Primate erythroparvovirus one* by the International Committee on Taxonomy of Viruses) [1]⁠ is the aetiological agent of *erythema infectiosum*, a common childhood disease. Given the tropism of B19V for erythroid progenitor cells it is also associated with a wide range of moderate to severe clinical manifestations related to anaemia, depending on the immunological and haematological status of the host [2]⁠. B19V primary infection can also occur in susceptible adults, and when it happens during pregnancy, it can be transmitted to the foetus through the placenta and trigger complications, including placental pathologies, miscarriage, foetal anaemia, hydrops, and even foetal death [3, 4]⁠. Up to 50 % of pregnant women may be susceptible to the virus, depending on the local circulation. B19V infection is endemo-epidemic; seasonal outbreaks are registered in late winter through early summer and epidemic peaks, every 4 to 6 years. Vertical transmission occurs in 25–50 % of cases and the risk of foetal damage is 2–18 %, depending on the series studied, the detection methods, and the occurrence of epidemic outbreaks [5–7]⁠.

The aetiological diagnosis is fundamental in the identification and follow-up of patients, as well as in deciding the administration of treatment. However, maternal infection is only suspected in approximately 40 % of cases. On the other hand, among adults, asymptomatic cases can be as high as 50 % (depending on the case definition), even during seasonal outbreaks, with a frequency of cases with manifestations meeting the classical presentation of B19V infection of less than 25 % [8–10]⁠. In particular, a study carried out to proactively follow B19V exposure and infection against the background of standard obstetric practice showed that 76 % of pregnant women with confirmed infection remained asymptomatic, contrasting to only 7 % non-pregnant women of child-bearing age. In this last group most B19V infections (93 %) presented with rash and arthralgia [10]⁠.

The aim of this report is to highlight the need and importance of optimizing early suspicion and detection of B19V infection during pregnancy in patients at risk.

## Case report

A 33-year-old woman, immunocompetent, gravida 2, para 1 (natural birth), with a followed-up pregnancy (five regular prenatal check-ups) of 22 weeks of gestation, consulted for intermittent contractions inappropriate for the gestational age, with 4 days of duration. She had also experienced an episode of fluid loss that lasted 3 h. At physical examination active foetal movements and normal foetal heartbeat values were observed. Other parameters evaluated did not evidence alterations in the evolution of pregnancy, including negative uterine dynamics, closed internal cervical os, negative amenorrhoea and negative genitorrhagia both at rest and valsalva. Stable vital signs were observed with normal levels of blood pressure. On superficial and deep palpation of the abdomen, it was soft, depressible and not painful with normal air-fluid noises and without resistance or peritoneal reaction. Ultrasound monitoring showed the presence of *hydrops fetalis* with foetal ascites and oedema of the subcutaneous cellular tissue (SCT), although amniotic fluid index (AFI) was within normal values (AFI 13). Immunological, cardiac and chromosomal causes were discarded, as well as other possible infectious causes, including Epstein Barr virus, Cytomegalovirus, syphilis and toxoplasmosis. Serological determination of anti-B19V IgM resulted positive in maternal serum, confirming an acute infection by this agent.

The following day, a Doppler ultrasonography revealed foetal anaemia (Doppler of the middle cerebral artery: 78 cm sec^−1^, representing 2.4 multiples of the median) and generalized hydrops, with pleural and pericardial effusion, oedema of the SCT>6 mm, and foetal heart failure. Placenta and amniotic fluid both appeared normal. Foetal haemoglobin (fHb) determined from umbilical cord blood evidenced severe anaemia (3 g dl^−1^).

In the next day, intrauterine blood transfusion was performed by channelling the umbilical cord at the level of the placental insertion. Foetal parameters at this time were: weight 530 g, blood type group A Rh positive, haemoglobin 1.6 g dl^−1^, haematocrit 4.7 %, erythrocyte sedimentation rate 134. Twenty-three cubic centimetres of inactivated, leukocyte-free blood, type 0 Rh negative, were transfused. After treatment, good tolerance was observed together with incipient recovery of fHb levels and haematocrit. Post-transfusion control showed fHb 6.3 g dl^−1^, haematocrit 18.7 %. The patient evolved without post-cordocentesis complications.

A second transfusion was scheduled 48 h after the first intervention, transfunding 23 cc of inactivated, leukocyte-free blood, type 0 Rh negative. During this procedure foetal bradycardia was observed and pericardial effusion occurred. Puncture was performed draining 4 cc of fluid. However, bradycardia persisted followed by foetal death. The mother evolved without post-cordocentesis complications. After labour induction, expulsion of the dead foetus occurred within 48 h, and the patient was subsequently discharged.

Two weeks later, a blood sample was taken from the patient for further serological studies. At this time, anti-B19V IgM was negative, while anti-B19V IgG was positive, as well as B19V DNA by end-point PCR. Furthermore, viral DNA remained detectable in two subsequent determinations in serum samples obtained at intervals of 6 months, totalling a detection period of 12 months since the diagnosis. In the next serum sample, after 18 months of foetal death, no viral DNA was detected. Viral loads were determined, remaining at the limit of detection while DNA was positive (Table 1). According to the registry in the clinical history, the patient remained asymptomatic throughout the gestation period prior to the episode of the consultation due to contractions. When revising the medical records, no classical signs of suspicion of B19V infection were noted -such as fever, rash, anaemia, or arthropathies. The only epidemiological risk factor was the presence of a school age-child at home (her son); there were no other remarkable risks of exposure to B19V infection (occupational, social or drug history).

**Table 1.** Immunological and virological markers of B19V infection in the pregnant patient. Follow-up to 18 months post primary infection.

Time of sample collection	Serum sample#	B19V DNA by end point PCR	Viral load by qPCR	IgM anti-B19V	IgG anti-B19V
22 wg	1	nd	nd	Positive*	nd
2 weeks after foetal death	2	Positive	1×10^3^ UI ml^−1^	Negative	Positive 28.5 UI ml^−1^
6 months after foetal death	3	Positive	1×10^3^ UI ml^−1^	Negative	Positive 67.5 UI ml^−1^
12 months after foetal death	4	Positive	<1×10^3^ UI ml^−1^	Negative	Positive 100.1 UI ml^−1^
18 months after foetal death	5	Negative	nd	Negative	Positive 90.1 UI ml^−1^

∗Biotrin immunofluorescence assay (the rest of the tests with ELISA RIDA Screen R-biopharm).

Nd, not determined; wg, weeks of gestation.

**Fig. 1.**
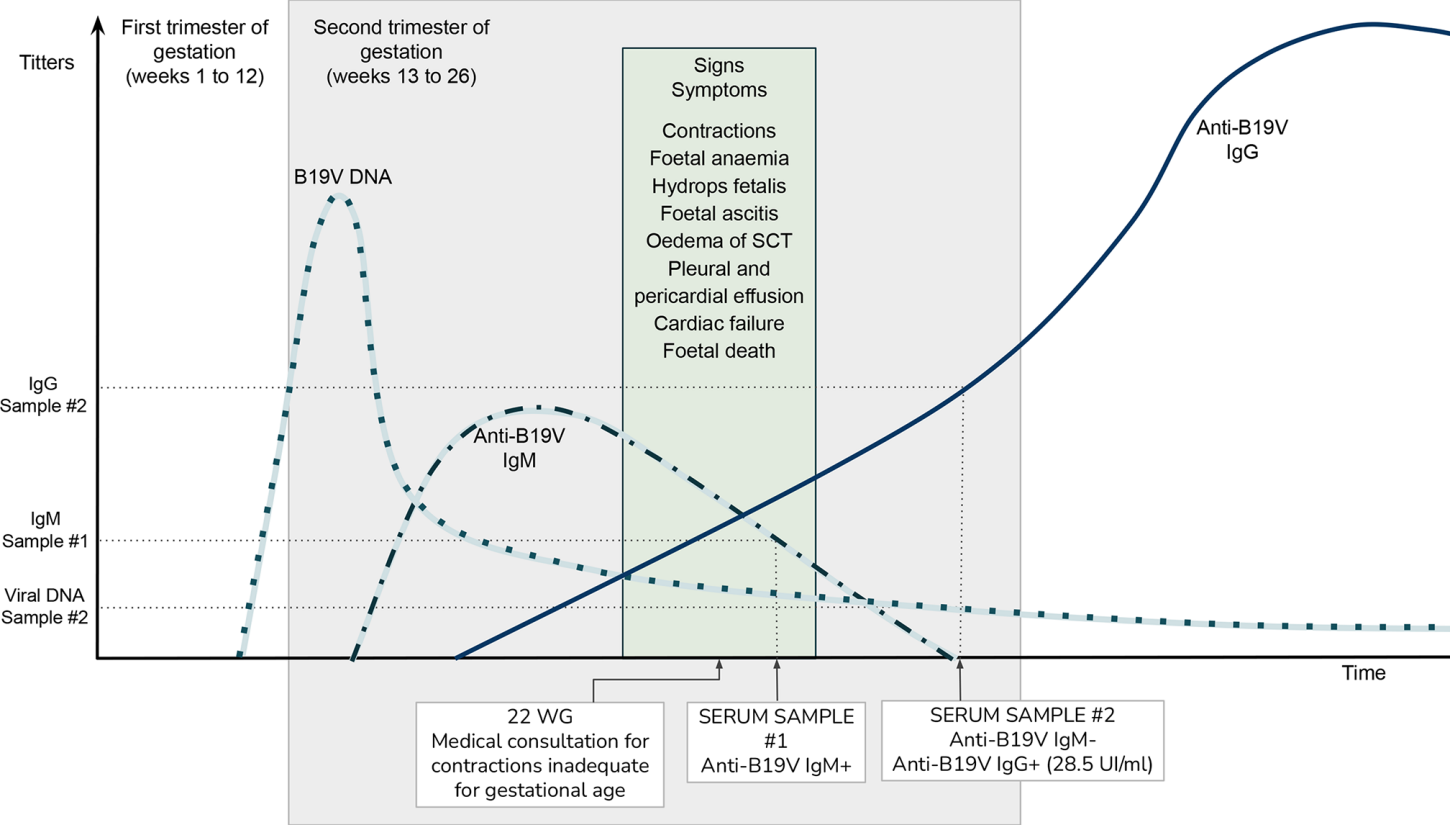
Hypothetical natural history of maternal infection for the case study. Results of serological and molecular markers are included. Infection might have started at some point between the end of the first trimester of pregnancy and the beginning of the second. After vertical transmission, intrauterine manifestations appeared, with the first clinical marker being inadequate contractions for the gestational age. At consultation foetal anaemia and generalized hydrops were observed with the final outcome of foetal death by the end of the second trimester of gestation. At this stage, the assay to determine specific IgM turned out negative, while levels of IgG were increasing and viral DNA remained detectable. WG: weeks of gestation.

Taking into account the previous data, we can hypothesize a possible course of the infection in this case as shown in Fig. 1.

## Discussion

In this report we present a case of B19V infection in a 33 year old pregnant woman, which was associated with an outcome of severe *hydrops fetalis,* foetal death and viral persistence in the patient for at least 12 months.

The pregnancy was at the 22nd week of gestation when the patient manifested intermittent inadequate contractions and fluid loss. During physical examination, no irregular parameters were observed with foetal movements and foetal heartbeats within normal ranges. B19V congenital infection was suspected based on ultrasound findings which revealed *hydrops fetalis* with ascitis and marked oedema of the SCT. There were no previous clinical signs that allowed physician’s early suspicion of maternal B19V infection. After discarding other possible causes considered in the differential diagnosis, ongoing B19V infection was confirmed by the presence of specific IgM in maternal serum.

When a B19V infection during pregnancy is suspected diagnosis first relies on serology in maternal serum, and specific IgM and IgG should be tested soon. In an immunocompetent individual, IgM becomes detectable within 10 days after transmission, while IgG levels rise up a few days after, conferring immunity for life [11]⁠. The presence of IgG and the absence of IgM at exposure may suggest previous immunity and no risk to the foetus -thus the importance of initial testing of IgG together with IgM to discard transmission. The detection of IgM indicates recent infection and an accurate pregnancy monitoring must be made, to evaluate foetal hydrops and predict foetal anaemia through Doppler measurement of the middle cerebral artery peak systolic velocity [12, 13]⁠. In our case, IgG was not determined in the first sample collected at the time of the mother’s consultation, in agreement to institution’s and current state guidelines. The positive IgM result, together with the other possible causes evaluated and discarded, as well as the complete clinical picture, were taken as sufficient confirmation of B19V as the aetiology in this case.

In a situation of contact with a person coursing the infection, a negative IgM result must be carefully interpreted since maternal serology within 7 days after exposure could still be negative. On the other hand, at the time that foetal signs are observed, it is possible that IgM levels in maternal serum may already have become low or even undetectable [14]⁠. Therefore, the determination of a single biomarker can lead to underestimating the aetiological diagnosis of B19V in congenital infections, especially if the diagnosis is based on determinations only in the maternal specimen, as it regularly occurs at the local level.

Regarding the acute infection, although the detection of specific IgM is confirmatory, it is recommended that diagnosis in pregnancy be based on a multi-factorial approach with the simultaneous detection of both immunological and virological parameters in serum from maternal peripheral blood [15]⁠. Determining B19V genome in maternal serum is advised, as it can improve the diagnostic accuracy and contribute to trace the stage of infection [16, 17]⁠.

In our case, critical foetal parameters such as low levels of fHb and haematocrit disclosed the severity of the clinical picture. After foetal death, serological and virological markers of B19V were assayed in the mother, up to 1 year and a half, with specimens collected at 6 months intervals. Two weeks after confirmatory diagnosis and foetal death, IgM was no longer detectable, while IgG levels showed a 3.5 fold-increase after 1 year. This kinetics of specific IgG, together with the initial detection of IgM, supports the diagnosis of a recent B19V infection. In addition, B19V DNA remained at low levels in two subsequent determinations, evidencing virus’ persistence in the patient. Finally, the infection was self-limited and the virus became undetectable 18 months after the acute phase of infection. Upon the first positive result by PCR, a follow-up was decided every 6 months on the advice of the treating medical team, given the patient’s concern. The risk of vertical transmission in a future pregnancy was discarded once viral DNA became negative.

The acute phase of infection is characterized by high viraemic load and the appearance of circulating specific antibodies is normally followed by a progressive clearance of viraemia [18]⁠. But among healthy blood donors it has been reported a detection rate of B19V DNA that varies from 0.02–21 % [19]⁠. In fact, it is now known that in a proportion of cases, after the acute phase of infection and mounting of the specific immune response, the viral genome remains detectable for weeks to several months or up to 2 years [20]⁠. Such B19V infections may result in asymptomatic persistence of virus in tissues [21]⁠. A single positive PCR result (DNAemia), without specific IgM and IgG accompanying data, is of difficult interpretation (or even misdiagnosing), as shown by Barlinn *et al*. [22]⁠. In their nested case-control study, they analysed B19V DNAemia in pregnant women with an outcome of foetal death and the controls were pregnancies that ended with live newborns. B19V PCR positivity was high and similar in both cases (24 %) and controls (28 %) and the vertical transmission rate was 9 % in cases and 12 % in controls [22]⁠. This also demonstrates the need for complete medical records (clinical and epidemiological data), when analysing diagnostic situations.

As mentioned, two intrauterine transfusions were performed in our case. Initially optimal response was observed; however, during the second transfusion, the foetal parameters worsened and the foetus did not survive. Currently, there are no specific treatments or vaccines available to treat life-threatening B19V infection, although there are preliminary *in vitro* studies that have demonstrated the effectiveness of some antiviral compounds [23, 24]⁠. In this scenario, early suspicion of B19V congenital infection becomes essential given the possibility to perform symptomatic treatments like intrauterine transfusion and, in highly compromised cases, the administration of intravenous immunoglobulin to the mother [25, 26]⁠.

Although B19V acute infection during pregnancy can cause severe foetal outcomes, routine serological screening is not indicated. B19V laboratory diagnosis is performed when / if maternal signs appear or when ultrasound reveals foetal abnormalities (*hydrops fetalis* and/or foetal anaemia). If the immune status of the patient for B19V is unknown, it is also strongly recommended to be tested under suspicion of exposure, a situation in which both IgM and IgG should be tested in an early maternal serum sample [15, 27, 28]⁠. Further testing in search of viral DNA may be required; in addition, tests in booked samples collected at previous check-ups may provide support for an accurate diagnosis [10]⁠. Thus, to improve diagnosis and management of confirmed cases, it is necessary to raise awareness among the healthcare workers for an early suspicion and increased testing. For that, epidemiological risk factors, especially exposure to children at home or work, the knowledge of the local seasonal/annual circulation of the virus by virological surveillance to identify periods of heightened community transmission [10]⁠, as well as a broad case definition -with clinical signs such as abnormal values of maternal anaemia [29]⁠- can be considered.

## Conclusions

We report a case of B19V infection in a pregnant woman diagnosed during the second trimester of gestation, with a fatal outcome for the foetus and persistence in the patient. Early diagnosis and knowledge of immune status, before or at the beginning of gestation, can contribute to improve the management of these cases. Early detection requires updating the diagnostic approach strategy in our patients at risk. In this group, suspecting and early diagnosis of infection could be achieved by considering epidemiological risk factors, exposure to exanthematous cases and a broad case definition.
